# Anthropometric failure and associated factors among children aged 6–23 months in Ethiopia

**DOI:** 10.1002/fsn3.3821

**Published:** 2023-11-16

**Authors:** Meseret Tamrat Gebretsadik, Assefa Legesse Sisay, Dessalegn Tamiru, Tefera Belachew

**Affiliations:** ^1^ Department of Nutrition and Dietetics, Public Health Faculty, Institute of Health Jimma University Jimma Ethiopia; ^2^ Department of Epidemiology, Public Health Faculty, Institute of Health Jimma University Jimma Ethiopia

**Keywords:** anthropometric failure, children aged 6–23 months, Ethiopia

## Abstract

Undernutrition in childhood is a crucial public health issue in Ethiopia. Yet, more than an assessment of undernutrition using conventional index is needed to conclude the overall prevalence of undernutrition among children aged 6–23 months. Therefore, this study aimed to assess the prevalence of undernutrition using composite index of anthropometric failure and its associated factors among children aged 6–23 months in Southwest Ethiopia. A community‐based cross‐sectional study was employed among 440 mother–child pairs selected using a two‐stage cluster sampling method in the rural Kersa district, Jimma Zone, Southwest Ethiopia. A pretested structured questionnaire was used to collect data. Multivariable logistic regression analysis was employed to identify factors associated with undernutrition. Variables with a *p*‐value of <.05 were considered statistically significant. The proportion of undernutrition using composite indexes of anthropometric failure was 57.3% among children aged 6–23 months. Children being male [AOR = 1.55; 95% CI (1.013, 2.373)], not met minimum acceptable diet (MAD) [AOR = 2.104; 95% CI (1.05, 4.214)], larger family size [AOR = 1.699; 95% CI (1.0791, 2.675)], having comorbidity [AOR = 3.31; 95% CI (2.068, 5.327)], and being in food insecurity household [AOR = 3.12; 95% CI (2.0, 4.868)] were more likely to be in anthropometric failure, whereas children from the mother who attended higher and above schooling [AOR = 0.244; 95% CI (0.093, 0.641)] were less likely to be in anthropometric failure. More than half of children aged 6–23 months were experienced anthropometric failure. Male children, those who have not received the MAD, come from larger families, have comorbidities, live in food‐insecure households, and have mothers with higher education levels were found to be at higher risk of anthropometric failure.

## Graphical abstract

Undernutrition in childhood is a significant public health problem in Ethiopia, particularly among children aged 6–23 months. This study aimed to assess the prevalence of undernutrition using composite indices of anthropometric failure and identify associated factors in the rural Jimma zone. The study found that 57.3% of children aged 6–23 months were undernourished, with factors such as being male, lower maternal education, not meeting the recommended minimum acceptable diet, larger family size, comorbidity, and food insecurity being significantly associated with undernutrition. More than half of children aged 6–23 months were experienced anthropometric failure.

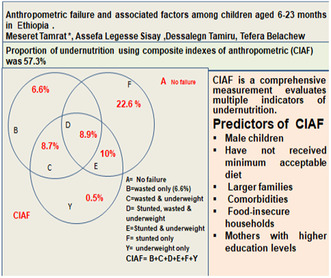

## 1 INTRODUCTION

Despite social and economic progress, there are still many people suffering from undernutrition worldwide (Black et al., 2013; Walker et al., 2007). All age groups are affected by undernutrition, but children under the age of two are the most at risk because of their need for more nutrients for rapid growth and development. According to a UNICEF report, there are 144 million stunted and 37 million wasting children under the age of five in the world (UNICEF et al., 2020).

The cause of undernutrition is multifactorial and varies across the region. Evidence showed that several factors associated with undernutrition are child age, sex of the child, maternal age, parental education and knowledge, comorbidity, family size, and food insecurity. Child feeding practices such as dietary diversity, meal frequency, and minimum acceptable diet (MAD) can lead to inadequate intake of essential nutrients, resulting in stunted growth, impaired cognitive development, and increased susceptibility to infections and diseases. It can have long‐term effects on their overall health and well‐being in infants and young children (Akombi et al., 2017; Bidira et al., 2021; Khanra et al., 2020; Kundu et al., 2022; Mahore, 2021).

Nutritional status in early childhood is crucial for long‐term health, strength, and mental development. It significantly impacts their overall well‐being, including their financial stability in their later life (Khanra et al., 2020; Kundu et al., 2022; Mahore, 2021).

The consequences of undernutrition ranged from short‐term mortality to long‐term disability and adulthood chronic diseases. Most of the damage associated with undernutrition occurs before children reach their second birthday (WHO, 2014).

The three conventional indicators (wasting, stunting, and underweight) are used to define undernutrition. Stunting (height for age) is the result of chronic and recurrent undernutrition, usually associated with recurrent infection and inappropriate feeding practices in early life that results in poor nutrient intake, absorption, and utilization. Stunting is a marker for poor child development and predicts poor cognitive and educational outcomes in later life. Wasting (weight for height) reflects acute malnutrition associated with recent illness failure or loss of child weight. Underweight (weight for age) is a composite measure of wasting and stunting (EPHI & ICF, 2019; UNICEF et al., 2020).

Even though Ethiopia has one of the world's fastest‐growing economies with a poverty line rate that declined from 55.5% to 26.7% (Gebru et al., 2018; Mohamed, 2017), undernutrition and poor child‐feeding practices remain an immense challenge among children under 5 years in the country (EDHS, 2016; EPHI, 2019; MOH, 2004). Based on the Ethiopia National Demographic Health Survey, the magnitude of stunting, wasting, and underweight was 37%, 7%, and 21%. There is also a higher regional difference in the prevalence of undernutrition (EPHI, 2019).

The separate analysis of child stunting, wasting, and underweight indexes cannot provide an accurate overall assessment of undernutrition burden in a population. Additionally, using reference information that does not accurately reflect the real problem contributes to the high prevalence of undernutrition. This is because comprehensive actions to tackle the issue have not been effectively implemented (Mahore, 2021; Permatasari & Chadirin, 2022).

Researchers have shown that conventional indicators are dependent entities due to the overlapping of these indexes. Hence, it is not enough to measure the overall prevalence of undernutrition (Svedberg, 2000), as some children may show signs of two or more anthropometric failures concurrently. Therefore, merely determining the frequency of undernutrition as a whole is insufficient.

The composite index of anthropometric failure (CIAF) was developed by Svedberg in 2000 and modified by Nandy et al. (2005) to give a single measure to estimate the overall prevalence of undernutrition.

In Ethiopia, earlier research on the prevalence of child undernutrition is excessively constrained, and they lack current data. In the past, some studies have evaluated child undernutrition using the conventional traditional three indexes, and others have evaluated undernutrition using just one composite index (CIAF) indicator. Conventional indexes may overlap when a single child exhibits two or more of them at once. Therefore, it is not enough to draw a conclusion on the prevalence of undernutrition in children. Additionally, no study establishes the relationship between CIAF and a composite index (MAD) of complementary feeding practices in Ethiopia and the study area. Therefore, this study aimed to determine the prevalence of undernutrition and its associated factors using a CIAF among children aged 6–23 months in the rural Jimma zone, Southwest Ethiopia. The current study's findings will help to strengthen the delivery of interventions to improve child nutrition status.

### 1.1 Key message

1. In Ethiopia and the research area, hardly any study evaluating anthropometric failure among children aged 6–23 months.
2. According to the current study, more than half of children aged 6–23 months were experienced anthropometric failure.
3. As a result, the findings of this study help the zone and regional health offices to better administer interventions to improve children's nutritional status by providing information on a single measure of undernutrition.

## 2 METHODS

### 2.1 Study area and period

A community‐based cross‐sectional study was conducted in the rural Kersa district, Jimma zone, Southwest Ethiopia from November to December 2021. Kersa district is 330 km away from the capital city of Ethiopia. The reliable rainfall ranges from 1200 to 2800 mL, and the altitude is between 1740 and 2660 meters above sea level. Among 26 kebeles (the smallest administration unit of the district in Ethiopia) in the district, four kebeles were semi‐urban and 22 rural kebeles. Based on the report of the respective districts' health offices in 2018, there were a 165,391 population with 83,579 males and 81,812 females. A total of 3651 children between the ages of 6 and 23 months were anticipated. The districts feature a midland agro‐ecological setting with a predominance of cereal and legume production, and some of these areas are growing chat (Daba, 2018).

### 2.2 Inclusion and exclusion criteria

Based on the criteria set for the interventional study, which includes mother–child pairs aged 6–23 months who were residents in the selected kebeles for more than 6 months and willing to participate were included. The exclusion criteria were mothers who were unable to communicate and excluded those children who received supplementation for the treatment of malnutrition.

### 2.3 Sample size determination and procedure

The anticipated sample size for the current cross‐sectional investigation, was estimated using G‐POWER software version 3.1.5, considering the upcoming interventional study with the assumptions of; 0.4 effect size between the intervention and control groups, a 5% level of significance (α 0.05; $Z_{\alpha /2}$ = 1.96; two‐tailed tests), and a power of 80% ($Z_{\beta }$ = 0.84). The estimated sample size for each group was 100 mother–child pairs. Including design effects of 2% and 10% non‐response rates, the total sample size was 440.

The two‐stage cluster sampling method was used to select the study participants. From 22 of the rural kebeles in the District based on the main agricultural production like grains and legumes, four kebeles were selected for our interventional study. Those selected kebeles consist of 25 Geres (the smallest administration unit of kebeles in Ethiopia) with a total population of 1194 children aged 6–23 months. Gere was considered as a cluster to select the study participants. From the total Geres, four adjacent Geres between the intervention and control arm were identified and excluded, and then from the remaining 21 Geres 14 (67%) were randomly selected. The sample size was then distributed proportionally according to the total number of children aged 6–23 months for each of the selected 14 Geres. Utilizing the family folder of the health extension workers, the study participants were selected using systematic sampling. The youngest child in a family with multiple children was considered.

#### 2.3.1 Study variables

Undernutrition (CIAF) is a dependent variable, and socio‐demographic factors, food insecurity, wealth index, maternal knowledge, decision‐making on household resources; child feeding practice, and child morbidity are independent variables.

### 2.4 Data collection tool, measurement, and procedures

A pretested structured questionnaire was used to collect socio‐demographic information, child feeding practice, anthropometric measurement, food insecurity, health, and related characteristics. Questionnaires were adapted from different literatures and EDHS (EPHI, 2019; Sabu et al., 2020; WHO, 2021). Ten graduate nurse data collectors and two Msc nutritionist supervisors were recruited and given them 3 days of intensive training about interview techniques, anthropometry measurements, and use of survey instruments.

### 2.5 Child feeding practice

Child feeding practices were collected using WHO (2021) standardized questionnaires based on the child's mother/caregiver's recall of food groups given to her child in 24 h before the data collection period. Then, all food that the child consumed was grouped into eight food groups: (1) grains, roots or tubers; (2) legumes and nuts; (3) vitamin A‐rich fruit and vegetable, (4) other fruits or vegetables, (5) flesh foods (meat, poultry, fish, organ meat); (6) eggs; (7) dairy products (milk, yoghurt, cheese); and (8) breast milk. Minimum dietary diversity (MDD) was computed by counting and summing the eight food groups. Minimum meal frequency (MMF) was calculated based on how many meals the children consumed in a 24‐h period. Minimum acceptable diet (MAD) was considered a composite indicator that relies on a combination of MDD and MMF from 24‐h recall (WHO, 2021).

Morbidity report was included if the child had been exposed to diarrhea cough, and fever in the past 2 weeks preceding the survey. If the mother said “yes” at least to one or more of these common morbidities, the child was considered as having the illness.

### 2.6 Anthropometric measurement

Ten experienced and trained BSc nurse conducted the anthropometric evaluation in accordance with standard practice. Weight was measured by UNICEF hanging spring scale to the nearest 0.1 kg with minimal clothing and length was measured in a recumbent position using the UNICEF portable length measuring board to the nearest 0.1 cm. The mother assisted in removing any extra clothing and shoes from the child before proceeding with the measurements. Afterward, the height board was positioned horizontally on a flat and leveled surface. The child's height (length) was then determined by having them lie on the board with feet together, knees straight, heels and buttocks making contact, shoulders relaxed, arms straight at the sides, and shoulder blades touching the length board.

To assess the variability of weight and height measurements, we calculated the coefficient of variation (CV) below 0.3, which is acceptable. Before weighing each child, the scales were calibrated to ensure reliability (Cashin & Oot, 2018; World Health Organization, 2019). The family folder card was used to determine the child age.

### 2.7 Composite index of anthropometric failure (CIAF)

The classification of the conventional indexes of children as stunted, wasted, and underweight was conducted based on their *Z*‐scores for weight‐for‐height (WHZ), length‐for‐age (LAZ), weight‐for‐age (WAZ), according to the World Health Organization (WHO, 2017) guideline. Children whose height for age *Z*‐scores, weight for age *Z*‐scores, and weight for height *Z*‐scores below minus two standard deviations (<−2 SD) from the median of the reference population are taken as stunted, underweight, and wasted, respectively (Cashin & Oot, 2018). The CIAF is the anthropometric index that combines the above three indexes (LAZ, WAZ, and WHZ) to estimate the overall prevalence of undernutrition as a single measure among children aged 6–23 months. The Nandy S model was used to calculate the overall prevalence of under nutrition. The classification of under nutrition based on CIAF was categorized into seven groups: Group A (no failure), Group B (wasted only), Group C (wasted and underweight), Group D (stunting, wasting, and underweight), Group E (stunting and underweight), Group F (stunted only), and Group Y (underweight only) (Bose, 2018; Nandy et al., 2005; Svedberg, 2000) (Table 1).

**TABLE 1:** Classification of composite index of anthropometric failure.

Groups	Description	Wasted	Stunted	Underweight
Group A	No failure	No	No	No
Group B	Wasted only	Yes	No	No
Group C	Wasted and underweight	Yes	No	Yes
Group D	Stunting, wasting, and underweight	Yes	Yes	Yes
Group E	Stunting and underweight	No	Yes	Yes
Group F	Stunting only	No	Yes	No
Group Y	Underweight only	No	No	Yes

Source: Adapted from Kutti (Bose, 2018).

### 2.8 Food insecurity

The Household Food Insecurity Access Scale (HFIAS), based on the Food and Nutrition Technical Assistance (FANTA) standards for developing countries, was used to assess household food insecurity. In terms of four Likert scale replies, the instrument's nine items, which are categorized as rarely (once or twice), occasionally (3–10), or often (>10 times), reflect the severity of food insecurity over the previous 4 weeks. The mothers/caregivers were expected to respond to these inquiries on behalf of the entire household. The cumulative score of the nine items ranged between 0 and 27, then the HFIAS score was categorized into four levels of household food insecurity: food secure, mild, moderate, and severe food insecurity (Coates et al., 2007). The Cronbach's alpha for the HFIAS in our study was 0.840, indicating high reliability or internal consistency scale.

### 2.9 Wealth index

The weights for the wealth index were created using principal components analysis based on data gathered on household assets, utilities, and other variables taken from the Ethiopian Demographic and Health Survey. The principal component analysis was used to develop latent factors representing wealth data in the first factor considered as the household wealth score. The scores were divided into five quintiles according to wealth: lowest, second, medium, fourth, and highest (EDHS, 2016).

### 2.10 Data quality

For the purpose of gathering data, the questionnaires were written in English and translated into Afan Oromo. Two weeks before to the actual data collection, a pre‐test was conducted on 5% of the questionnaires distributed outside the study area. Three days of intensive training on the data collection process were given for 10 graduate nurse for data collectors and two Msc nutritionist supervisors. These data collectors and supervisors were fluent in the local language. To confirm the accuracy of the data, two supervisors filled out daily questionnaires and examined the data collection procedure.

### 2.11 Data analysis

Epi Data Manager Version 4.6 was used to enter the data, which was then exported to SPSS Version 25 for analysis. The data were summarized using descriptive statistics, and the findings were displayed using frequency, tables, texts, and figures. Histograms were used to verify if continuous variables were normal. To develop nutritional indexes, anthropometric data were converted from SPSS to WHO Anthro software version 3.2.2. Bivariate logistic regression analysis was utilized to identify the candidate variables for multivariable analysis and variables with a *p*‐value less than .25 were added to a multivariable logistic regression. The significance and strength of association were determined using *p*‐value of ≤.05, and adjusted odds ratio (AOR) with a 95% confidence interval (CI), respectively. The variance inflation factor (VIF) values for each variable were less than 1.0, indicating that multi‐collinearity was not a problem in the model. The Hosmer–Lemeshow test was used to assess the goodness of fit of the model (*p*‐value: .782).

### 2.12 Ethical clearance

The ethical clearance was obtained from the Institutional Review Board (IRB) of Jimma University, Institute of Health, Faculty of Public Health, with approval number (ref. HRPGC/536/2020). All mothers or caretakers involved in the study were informed about the purpose and told they could stop the interview at any time they wanted.

## 3 RESULTS

### 3.1 Sociodemographic characteristics of the study participants

In this study, 438 child–mother pairs were participated with a response rate of 99.4%. Females made up more than half of the children (52.7%). Only 171 (39.5%) mothers or caregivers had a formal education, and 400 (92.2%) of the mothers were housewives. Mothers were overwhelmingly Muslim (99.3%) and Oromo (98.4%) by ethnicity. Almost half of the participants (49.3%) had more than five family members, and 424 of the participants (96.8%) were married. Nearly half of the household's resources (41.1%) were under the authority of other family members, followed by child fathers (26.0%) and mothers alone (14.2%). Only 18.7% of women and fathers, however, controlled the household's resources. In terms of food security, 66.0% of households were food insecure, while 6.4% were severely food insecure. The second and fourth wealth index quintiles were represented by 122 (27.9%) and 101 (23.1%) of the respondents, respectively (Table 2).

**TABLE 2:** Socio‐demographic characteristics of the mothers/caretakers and their children in rural of Jimma zone, southwest Ethiopia, 2021 (*N* = 438).

Variables (*N* = 440)	Category	Frequency	Percent
Child age in month	6–11	303	69.2
	12–17	121	27.6
	18–23	14	3.2
Sex of child	Male	207	47.3
	Female	231	52.7
Mother age in years	< 25	232	53.0
	25–34	154	35.1
	>35	52	11.9
Mother education	Informal education	267	61.0
	Primary school	146	33.3
	Secondary & above	25	5.7
Mother occupation	Housewife	404	92.2
	Farmer	16	3.7
	Governmental employee	4	0.9
	Others^a^	14	3.2
Religion	Muslim	435	99.3
	Orthodox	2	0.5
	Protestant	1	0.2
Ethnicity	Oromo	431	98.4
	Others^b^	7	1.6
Marital status	Single	5	1.1
	Married	424	96.8
	Widowed	5	1.1
	Separated	4	1.0
Decision making on household resource	Jointly^c^	82	18.7
	Mother only	62	14.2
	Father only	114	26.0
	Family^d^	180	41.1
Family size	>5	222	50.7
	<5	236	49.3
Wealth index	Lowest	79	18.0
	Second	122	27.9
	Middle	50	11.4
	Fourth	101	23.1
	Highest	86	19.6
Food insecurity	Food secured	149	34.0
	Mild FI	189	43.2
	Moderate food insecurity	72	16.4
	Severe food insecurity	28	6.4

^a^ Merchant, private.

^b^ Amhara, Dawero, Kaffa.

^c^ Both mothers and fathers.

^d^ Mother‐in‐law, other both spouse family.

### 3.2 Feeding practice and health‐related characteristics

In the previous 24 h, nearly all mothers (98.4%) breastfed their children. Almost half of mothers and caregivers (47.9%) began complementary feeding at 6 months, and 46.1% bottle‐fed their children. In terms of nutritional diversity and meal frequency, 14.4% and 64.8% of children received the recommended amounts. However, only 46 children (10.7%) met the recommended MAD. Two hundred eighty‐seven (65.5%) children were born in hospitals. In terms of illness, 34.5% of children had been sick in the previous 15 days. Coughing was experienced by nearly half of the children (47.7%), followed by diarrhea (43.7%). Only 5.7% of mothers prepared cereal‐based complementary foods using traditional household food processing techniques (THHFP). Nearly half (52%) of the 25 (5.7%) mothers acquired information about THHFP from their mother‐in‐law and neighbors (28%) (Table 3).

**TABLE 3:** Feeding practice and health‐related characteristics among 6–23 months of children in rural Jimma zone, southwest Ethiopia, 2021 (*N* = 438).

Variable	Category	Frequency	Per cent
Ever breastfed (*N* = 438)	Yes	431	98.4
	No	7	1.6
Ever bottlefed (*N* = 438)	Yes	202	46.1
	No	236	53.9
Initiation of complementary foods	Not started	7	1.6
	<6 month	119	27.2
	At 6 month	210	47.9
	>6 month	102	23.3
Minimum dietary diversity	Met	63	14.4
	Not met	375	85.6
Minimum meal frequency	Met	284	64.8
	Not met	154	35.2
Minimum acceptable diet	Met	47	10.7
	Not met	391	89.3
Place of delivery	Health facility	287	65.5
	Home	151	34.5
Practice of THHFP technique	Yes	25	5.7
	No	413	94.3
Information about THHFP technique	Mother‐in‐law	13	52.0
	Neighbors	7	28.0
	Health workers	3	12.0
	Mass media	2	8.0
Illness	Yes	151	34.5
	No	287	65.5
Type of illness (*N* = 159)	Diarrhea	66	43.7
	Cough	72	47.7
	Intestinal parasite	11	7.3
	Others^a^	2	1.3

Abbreviation: THHFP, Traditional household food processing.

^a^ Fever, vomiting, ear infection.

### 3.3 Assessment of under nutrition using conventional and composite index anthropometric failure

Table 4 presented the prevalence of undernutrition based on the composite index anthropometric failure (CIAF) classification by age and sex among children aged 6–23 months. The overall proportion of anthropometric failure was 57.3%. Wasted only (6.6%), wasted and underweight (8.7%), stunted, wasted and underweight (8.9%), stunting and underweight (10%), stunted only (22.6%), and underweight only (0.5%) was accounted to anthropometric failure. Among the subgroups of CIAF (Groups A to Y) by a specific age, stunting only (Group F) was the highest (23%), and underweight only (Group Y) was the lowest (0.6%) among children aged 6–12 months. In contrast, wasted and stunted, and underweight (Group D) was the highest (20.5%), and none of the children faced underweight only (Group Y) among 13–23 months of children. The proportion of CIAF subgroups by specific sex, stunting only (Group F), was ranked high among females (24.2%) and males (22.2%). Underweight only (Group Y) was lower in males, and no one experienced underweight only among females (Table 4).

**TABLE 4:** Prevalence of undernutrition based on the CIAF classification by age and sex among 6–23 months of children in rural areas of Jimma Zone, Southwest Ethiopia, 2021 (*N* = 438).

Categories of CIAF	Age group in months		Sex		Total
	6–12	13–23	Male	Female	
No failure (A)	155 (43.7)	32 (38.6)	76 (36.7)	111 (48.1)	187 (42.7)
Wasting only (B)	27 (7.6)	2 (2.4)	13 (6.3)	16 (6.9)	29 (6.6)
Wasting & underweight (C)	33 (9.3)	5 (6.0)	22 (10.6)	16 (6.9)	38 (8.7)
Wasting, stunting & underweight (D)	22 (6.2)	17 (20.5)	24 (11.6)	15 (6.5)	39 (8.9)
Stunting & underweight (E)	32 (9.0)	12 (14.5)	27 (13.0)	17 (7.4)	44 (10.0)
Stunting only (F)	84 (23.7)	15 (18.1)	43 (20.8)	56 (24.2)	99 (22.6)
Underweight only (Y)	2 (0.6)	0 (0.0)	2 (1.0)	0 (0.0)	2 (0.5)
CIAF = B + C + D + E + F + Y	200 (56.3)	51 (61.4)	131 (63.3)	120 (51.9)	251 (57.3)

Abbreviation: CIAF, Composite index of anthropometric failure.

The prevalence of conventional indexes by age and gender is shown in Table 5. It is observed that for all three measures of malnutrition, the prevalence decreases as the child gets older. The proportion of wasting is higher (28.9%) in children 12–23 months compared to children aged 6–12 months. The prevalence of stunting is higher in children aged 13–24 months (53.0%) compared to children aged 6–12 months (38.9%). As same, prevalence of underweight is higher among children aged 13–24 months (41%) compared to children aged 6–12 months. Specifically, the percentage of wasted, stunted, and underweight males is higher than that of females. The prevalence of wasting is higher in males (28.5%) compared to females (20.3%). Similarly, in stunting, males having a higher prevalence (45.4%) compared to females (38.1%). In underweight, males having a higher prevalence (36.2%) compared to females (20.8%). In comparison, conventional measures of wasting, stunting, and underweight were 24.2%, 41.6%, and 28.1%, respectively, all of which were lower than the CIAF (Table 5).

**TABLE 5:** Prevalence of undernutrition using conventional indexes by age and sex among 6–23 months of children, 2021.

Conventional indexes	Child age in months		Sex		Total
	6–12	13–23	Male	Female	
Wasted (Weight for height)	82 (23.1)	24 (28.9)	59 (28.5)	47 (20.3)	106 (24.2)
Stunted (Height for age)	138 (38.9)	44 (53.0)	94 (45.4)	88 (38.1)	182 (41.6)
Underweight (Weight for age)	89 (25.1)	34 (41.0)	75 (36.2)	48 (20.8)	123 (28.1)

### 3.4 Factors associated with CIAF among children 6–23 months in rural Jimma zone, southwest Ethiopia, 2019

On bivariate analysis, the factors found to be significantly associated with the overall prevalence of under nutrition using CIAF (Anthropometric failure) were the sex of the child, mother's education, MAD, birth order, family size, illness within 2 weeks and HH food security status.

After controlling the potential confounders, in the final multivariable logistic regression, male children had 1.5 times higher odds of CIAF (AOR = 1.55, 95% CI: 1.013–2.373) as compared to female children. Those children whose mothers attained secondary and above schooling were 75% less likely (AOR = 0.244, 95% CI: 0.093–0.641) to be undernourished compared to children whose mothers had formal education. A child who did not met MAD was 2.0 times more likely malnourished than those who had met the recommended MAD (AOR = 2.14, 95% CI: 1.065–4.301). Children who lived in a family of more than five members were 1.6 times more likely to have an anthropometric failure than children with less than five family members (AOR = 1.699, 95% CI: 1.079–1.675). Children who experienced illness in the past 2 weeks were 3.3 times more likely (AOR = 3.31, 95% CI: 2.068–5.327) to have anthropometric failure than those who did not have the disease. Children from food‐insecure households were 3.12 times more likely to have an anthropometric failure (AOR = 3.2, 95% CI: 2.00–4.863) compared to food‐secure household counterparts (Table 6).

**TABLE 6:** Multivariable logistic regression of factors associated with composite indexes of anthropometric failure among children aged 6–23 months in rural Jimma zone, southwest Ethiopia, 2021 (*N* = 438).

Variables (*n* = 438)	CIAF		COR (95% CI)	AOR (95% CI)
	Failure	No failure		
Child sex				
Male	131 (63.3)	76 (36.7)	1.59 (1.087–2.338)*	1.55 (1.013–2.373)*
Female	120 (51.9)	111 (48.1)	Ref.	Ref.
Mother education				
Informal	168 (62.9)	99 (37.1)	Ref.	Ref.
Primary	75 (51.4)	71 (48.6)	0.622 (0.414–0.337)	0.709 (0.448–1.120)
≥Secondary	8 (32.0)	17 (68.0)	0.277 (0.115–0.666)*	0.244 (0.093–0.641)*
MAD				
Met	18 (38.3)	29 (61.7)	Ref.	Ref.
Not met	233 (59.6)	158 (40.4)	2.37 (1.276–4.425)*	2.104 (1.05–4.214)*
Birth order				
1	31 (50.9)	30 (49.2)	Ref.	Ref.
2–4	122 (52.6)	110 (47.40)	1.073 (0.610–1.887)	0.953 (0.50–1.816)
>5	99 (67.6)	47 (32.4)	2.019 (1.096–3.716)*	1.652 (0.806–3.387)
Family size				
<5	111 (50.0)	111 (50.0)	Ref.	Ref.
>5	140 (64.8)	76 (35.2)	1.84 (1.255–2.704)*	1.699 (1.079–2.675)*
Comorbidity^a^				
Yes	111 (44.2)	40 (21.4)	2.914 (1.897–4.476)	3.31 (2.068–5.327)**
No	140 (55.8)	147 (78.6)	Ref.	Ref.
Food security status				
Secured	58 (38.9)	91 (61.1)	Ref.	Ref.
Not secured	193 (66.6)	96 (33.2)	3.154 (2093–4754)**	3.12 (2.0–4.868)**

Abbreviation: MAD, Minimum acceptable diet.

^a^ Diarrhea cough, and fever: Ref. indicates for the reference category of variables; Hosmer and Lemeshow test = 0.782.

*p‐value < .05; **p‐value < .001.

## 4 DISCUSSION

In the current study, a single composite index (CIAF) was computed from the three conventional anthropometric indexes (wasting, stunting, and underweight), as conventional indexes cannot determine an aggregate estimate of undernutrition among infants and young children. Thus, the current finding revealed that the prevalence of CIAF was (57.3%), and it was higher than the conventional index of wasting (24.2%), stunting (41.6%), and underweight (28.1%). The current study's findings on conventional indexes of wasting, stunting, and underweight were higher than those of the Ethiopian national demographic health survey (EPHI & ICF, 2019).

Children who are older and being a male are more affected by undernutrition in both conventional and composite indexes, which is consistent with prior studies in Ethiopia (EPHI, 2019; Fenta et al., 2021; Nigusu et al., 2019). This could be due to their higher dietary requirements for growth and development at this age, as well as increased exposure to infection. Furthermore, males had a higher rate of malnutrition than girls, which could be attributed to increased energy requirements, social and cultural norms favoring female children, and limited access to healthcare and resources.

More than half of the children in this study (57.3%) had CIAF, which was substantially similar to Pakistan's (52.2%) and, India's findings (54.4%) (Balogun et al., 2021; Goswami, 2016; Kundu et al., 2022). However, several research studies found that the prevalence of undernutrition (CIAF) is lower in Ethiopia from 46.2% to 50.8% (Bidira et al., 2021; Endris et al., 2017; Fenta et al., 2021; Kassie & Workie, 2020), in Malawi (50.6%) (Ziba et al., 2018), Indonesia (42.1%) (Permatasari & Chadirin, 2022), and Bangladesh (48.3%) (Islam & Biswas, 2020). This disparity could be attributed to differences in study duration, feeding practices, information gaps, and socioeconomic and cultural factors. Furthermore, the majority of the preceding research was conducted on various age groups: children under the age of five and preschool children. The higher prevalence of CIAF found in this study could be attributed to the fact that children in their first 2 years are more vulnerable to undernutrition than toddlers or preschool children due to the high demand for nutrients to support their rapid growth and development, poor child feeding practices, and infection. Furthermore, the current research area's population is reliant on cereal and grain cultivation. These factors may result in a lack of variety in a child's diet, putting children at risk of undernutrition.

However, the proportion of CIAF was lower than the finding from Yemen (70%) (Al‐Sadeeq et al., 2018), India 62% (Gupta et al., 2017), and Bhagwah in India (67.5%) (Mahore, 2021). This discrepancy could be attributed to a difference in the study period, which was conducted earlier than the current study.

According to this study, mother's higher education was associated with CIAF. Children whose mothers had completed secondary school or above were less likely to have CIAF than children whose mothers had never completed formal education. It is consistent with prior research from Ethiopia (Endris et al., 2017; Fenta et al., 2021; Seboka et al., 2021), Pakistan (Balogun et al., 2021), Bangladesh (Das et al., 2022), and Bengal (Kundu et al., 2022) which found that when mothers' educational status improves, the probability of their children failing to anthropometric failure decreases. These clearly emphasize that, depending on their educational level, educated women may receive direct information about their child's diet and nutritional status.

This study found a significant association between CIAF and larger family size, which is consistent with research done in Ethiopia (Bidira et al., 2021). The reason could be that large families are more likely to have financial difficulties and are therefore unable to give their children a balanced and nutritious food that would support normal growth and development. The current study found that illness in the previous 2 weeks (cough, fever, and intestinal parasites) is a predictor of CIAF. Likewise, research from Ethiopia and India consistently found an association between CIAF and illness (Agarwal et al., 2015; Bidira et al., 2021; Fenta et al., 2021; Kassie & Workie, 2020). This resemblance could be explained by the vicious cycle of illness and nutrition. Infection can cause a loss of appetite, poor digestion, nutrient malabsorption, and a weaker immune system. As a result, it may jeopardize children's nutritional status (Chiabi et al., 2018).

Male children were 1.5 times more likely than females to experience anthropometric failure, according to our findings. This outcome is consistent with previous research from Ethiopia and India (Bidira et al., 2021; Fenta et al., 2021; Kassie & Workie, 2020). One possible explanation is that male children are more influenced by environmental variables and food than girls in their early years (Wells, 2000).

To the best of our knowledge, this is the first study in Ethiopia that has determined the association of a MAD with CIAF. In the current study, children aged 6–23 months who did not receive the recommended MAD were more likely to experience CIAF than children who did. We were unable to compare our findings to other studies since no studies determining the association of MAD with CIAF were found. However, our findings are comparable to research in Nigeria that used conventional measures to quantify undernutrition and showed an association between MAD and underweight and stunting but not wasting (Udoh & Amodu, 2016; Ziba et al., 2018). This could be due to insufficient nutritional intake, and the quality of supplemental foods raises the risk of chronic malnutrition. Dietary diversity and MMF index, respectively, are predictors of child linear growth and proxies for energy intake. As a result, MAD is a composite index of dietary diversity and minimal meal frequency used to capture multiple dimensions of feeding in order to promote optimal growth and development of children, and it may play a role in undernutrition (Akombi et al., 2017; INDDEX, 2018; WHO, 2009).

This finding showed that children who lived in food‐insecure households were three times more likely exposed to anthropometric failure compared to their counterparts of children in food‐secure households. The finding is similar to India and Rwanda studies (Agho et al., 2019; Sabu et al., 2020). It could be due to the low consumption and monotonous diet in food insecure households that may deprive adequate intake of essential nutrients and can affect the nutritional status of children. The South Africa study reported that household food insecurity is the primary contributor to undernutrition among children (Ntila et al., 2017).

### 4.1 Strength of the study

This study considered MAD as a predictor variable. A MAD is a composite index and a proxy for the quality of complementary food, and its effect may reflect on a child's nutritional status. Thus, this study showed the association between MAD and CIAF.

### 4.2 Limitation

The possible limitations of this study might be recall bias and social desirability bias as the data rely on self‐report. The nature of the cross‐sectional study limits the ability to conclude the cause‐and‐effect relationship between CIAF and independent variables.

## 5 CONCLUSION

In conclusion, more than half of the children aged 6–23 months in the study area were found to have anthropometric failure, as measured by the CIAF. The study found that being male, having a mother with higher education, not having a MAD, having a larger family size, having comorbidities, and experiencing food insecurity were all predictors of anthropometric failure. CIAF is a composite measure that considers all types of undernutrition to provide a comprehensive assessment. These findings provide better evidence that CIAF is an effective tool for identifying undernutrition in children aged 6–23 months. The results of this study can be used to develop targeted interventions to reduce undernutrition. Policymakers and researchers are also urged to utilize CIAF to accurately assess children's nutritional status in future studies and policy‐making decisions.

## AUTHOR CONTRIBUTIONS

**Meseret Tamrat Gebretsadik:** Conceptualization (lead); data curation (lead); formal analysis (lead); funding acquisition (lead); investigation (lead); methodology (lead); project administration (lead); resources (lead); software (lead); supervision (lead); validation (lead); visualization (lead); writing – original draft (lead); writing – review and editing (equal). **Assefa Legesse Sisay:** Formal analysis (equal); methodology (equal); software (equal); visualization (equal); writing – review and editing (equal). **Dessalegn Tamiru:** Conceptualization (equal); data curation (equal); formal analysis (equal); funding acquisition (equal); investigation (equal); methodology (equal); project administration (equal); resources (equal); software (equal); supervision (equal); validation (equal); visualization (equal); writing – review and editing (equal). **Tefera Belachew:** Conceptualization (equal); data curation (equal); formal analysis (equal); funding acquisition (equal); investigation (equal); methodology (equal); project administration (equal); resources (equal); software (equal); supervision (equal); validation (equal); visualization (equal); writing – review and editing (equal).

## CONFLICT OF INTEREST STATEMENT

The authors declare no conflicts of interest.

## ETHICS STATEMENT

This study was approved by the Institutional Review Board (IRB) of Jimma University.

## INFORMED CONSENT

Written informed consent was obtained from all study participants.

## Data Availability

The data that support the findings of this study are available on request from the corresponding author.

## References

[fsn33821-bib-0001] Agarwal, D. , Misra, S. K. , Chaudhary, S. S. , & Prakash, G. (2015). Are we underestimating the real burden of malnutrition? An experience from community‐based study. Indian Journal of Community Medicine, 40(4), 268–272. 26435601 10.4103/0970-0218.164401 PMC4581148

[fsn33821-bib-0002] Agho, K. E. , Mukabutera, C. , Mukazi, M. , Ntambara, M. , Mbugua, I. , Dowling, M. , & Kamara, J. K. (2019). Moderate and severe household food insecurity predicts stunting and severe stunting among Rwanda children aged 6–59 months residing in Gicumbi district. Maternal & Child Nutrition, 15(3), e12767. 30548790 10.1111/mcn.12767 PMC7198954

[fsn33821-bib-0003] Akombi, B. J. , Agho, K. E. , Hall, J. J. , Wali, N. , Renzaho, A. M. , & Merom, D. (2017). Stunting, wasting and underweight in sub‐Saharan Africa: A systematic review. International Journal of Environmental Research and Public Health, 14(8), 863. 28788108 10.3390/ijerph14080863 PMC5580567

[fsn33821-bib-0004] Al‐Sadeeq, A. H. , Bukair, A. Z. , & Al‐Saqladi, A.‐W. M. (2018). Assessment of undernutrition using composite index of anthropo‐metric failure among children aged <5 years in rural Yemen. Eastern Mediterranean Health Journal, 24(12), 1119–1126. 10.26719/2018.24.12.1119 30799551

[fsn33821-bib-0005] Balogun, O. S. , Asif, A. M. , Akbar, M. , Chesneau, C. , & Jamal, F. (2021). Prevalence and potential determinants of aggregate anthropometric failure among Pakistani children: Findings from a community health survey. Children, 8(11), 1010. 34828722 10.3390/children8111010 PMC8622924

[fsn33821-bib-0006] Bidira, K. , Tamiru, D. , & Belachew, T. (2021). Anthropometric failures and its associated factors among preschool‐aged children in a rural community in southwest Ethiopia. PLoS One, 16(11), e0260368. 34843555 10.1371/journal.pone.0260368 PMC8629177

[fsn33821-bib-0007] Black, R. E. , Victora, C. G. , Walker, S. P. , Bhutta, Z. A. , Christian, P. , de Onis, M. , Ezzati, M. , Grantham‐McGregor, S. , Katz, J. , Martorell, R. , Uauy, R. , & Maternal and Child Nutrition Study Group . (2013). Maternal and child undernutrition and overweight in low‐income and middle‐income countries. The Lancet, 382(9890), 427–451. 10.1016/S0140-6736(13)60937-X 23746772

[fsn33821-bib-0008] Bose, K. (2018). The concept of composite index of anthropometric failure (CIAF): Revisited and revised. Anthropology, 3, 32–35.

[fsn33821-bib-0009] Cashin, K. , & Oot, L. (2018). A practical tool for program planners, managers, and implementers (guide to anthropometry) . Food and Nutrition Technical Assistance III Project/FHI.

[fsn33821-bib-0010] Chiabi, A. , Obadeyi, B. , Nguefack, F. , Chiabi, R. , Berinyuy, E. , Chiabi, E. , Mbang, T. A. , & Obama, M. (2018). The vicious cycle of malnutrition and childhood infections – What are the policy implications? Archives of Pediatrics and Neonatology, 1(1), 21–25.

[fsn33821-bib-0011] Coates, J. , Swindale, A. , & Bilinsky, P. (2007). Household Food Insecurity Access Scale (HFIAS) for measurement of food access: Indicator guide: version 3 .

[fsn33821-bib-0012] Daba, M. (2018). Agro climatic characterization in the selected woredas of western Oromia, Ethiopia. Journal of Earth Science & Climatic Change, 9(455), 2.

[fsn33821-bib-0013] Das, R. , Kabir, M. F. , Ashorn, P. , Simon, J. , Chisti, M. J. , & Ahmed, T. (2022). Maternal underweight and its association with composite index of anthropometric failure among children under two years of age with diarrhea in Bangladesh. Nutrients, 14(9), 1935. 35565901 10.3390/nu14091935 PMC9105738

[fsn33821-bib-0014] EDHS . (2016). Addis Abeba: Central statistical authority of Ethiopia and Ministry of Health .

[fsn33821-bib-0015] Endris, N. , Asefa, H. , & Dube, L. (2017). Prevalence of malnutrition and associated factors among children in rural Ethiopia. BioMed Research International, 2017, 6587853. 28596966 10.1155/2017/6587853 PMC5449753

[fsn33821-bib-0016] EPHI . (2019). Ethiopia mini demographic and health survey 2019: Key indicators .

[fsn33821-bib-0017] EPHI , & ICF . (2019). Ethiopia mini demographic and health survey 2019 .

[fsn33821-bib-0018] Fenta, H. M. , Zewotir, T. , & Muluneh, E. K. (2021). Disparities in childhood composite index of anthropometric failure prevalence and determinants across Ethiopian administrative zones. PLoS One, 16(9), e0256726. 34555038 10.1371/journal.pone.0256726 PMC8459952

[fsn33821-bib-0019] Gebru, M. , Remans, R. , Brouwer, I. D. , Baye, K. , Melesse, M. B. , Covic, N. , Habtamu, F. , Abay, A. H. , Hailu, T. , Hirvonen, K. , Kassaye, T. , Kennedy, G. , Lachat, C. , Lemma, F. , McDermott, J. , Minten, B. , Moges, T. , Reta, F. , Tadesse, E. , … van den Berg, M. (2018). Food systems for healthier diets in Ethiopia: Toward a research agenda . IFPRI Discussion Paper.

[fsn33821-bib-0020] Goswami, M. (2016). Prevalence of under‐nutrition measured by composite index of anthropometric failure (CIAF) among the Bhumij children of Northern Odisha, India. Journal of Nepal Paediatric Society, 36(1), 61–67.

[fsn33821-bib-0021] Gupta, G. , Sharma, A. K. , & Choudhary, T. S. (2017). Assessment of undernutrition among children below 5, using composite index of anthropometric failure (CIAF). Indian Journal of Community Health, 29(1), 108–113.

[fsn33821-bib-0022] INDDEX . (2018). Data4Diets: Building blocks for diet‐related food security analysis . Tufts University.

[fsn33821-bib-0023] Islam, M. S. , & Biswas, T. (2020). Prevalence and correlates of the composite index of anthropometric failure among children under 5 years old in Bangladesh. Maternal & Child Nutrition, 16(2), e12930. 31867876 10.1111/mcn.12930 PMC7083426

[fsn33821-bib-0024] Kassie, G. W. , & Workie, D. L. (2020). Determinants of under‐nutrition among children under five years of age in Ethiopia. BMC Public Health, 20(1), 1–11. 32220224 10.1186/s12889-020-08539-2 PMC7099779

[fsn33821-bib-0025] Khanra, P. , Chakraborty, R. , & Bose, K. J. (2020). Factors affecting anthropometric failure in urban Bengalee children of Purba Medinipur, West Bengal, India. Human Biology Review, 9(4), 309–327.

[fsn33821-bib-0026] Kundu, R. N. , Hossain, M. G. , Haque, M. A. , Biswas, S. , Huq, M. M. , Pasa, M. K. , Sabiruzzaman, M. , & Bharati, P. (2022). Factor associated with anthropometric failure among under‐five Bengali children: A comparative study between Bangladesh and India. PLoS One, 17(8), e0272634. 35930584 10.1371/journal.pone.0272634 PMC9355208

[fsn33821-bib-0027] Mahore, J. (2021). Nutritional assessment using composite index of anthropometric failure (CIAF) among school‐going children in J&K, India. Journal of Tianjin University Science and Technology, 54, 405–420.

[fsn33821-bib-0028] MOH . (2004). National strategy for infant and young child feeding .

[fsn33821-bib-0029] Mohamed, A. A. (2017). Food security situation in Ethiopia: A review study. International Journal of Health Economics and Management, 2(3), 86–96.

[fsn33821-bib-0030] Nandy, S. , Irving, M. , Gordon, D. , Subramanian, S. , & Smith, G. D. (2005). Poverty, child undernutrition and morbidity: New evidence from India. Bulletin of the World Health Organization, 83, 210–216. 15798845 PMC2624218

[fsn33821-bib-0031] Nigusu, D. , Kemal, F. , & Betela, B. (2019). Determinants of malnutrition among under‐five children: A case of ARSI zone selected woredas in Oromia Regional State, Ethiopia. Journal of Advances in Medicine and Medical Research, 28(8), 1–11.

[fsn33821-bib-0032] Ntila, S. , Siwela, M. , Kolanisi, U. , Abdelgadir, H. , & Ndhlala, A. (2017). An assessment of the food and nutrition security status of weaned 7–12 months old children in rural and peri‐urban communities of Gauteng and Limpopo Provinces, South Africa. International Journal of Environmental Research and Public Health, 14(9), 1004. 28862694 10.3390/ijerph14091004 PMC5615541

[fsn33821-bib-0033] Permatasari, T. A. E. , & Chadirin, Y. (2022). Assessment of undernutrition using the composite index of anthropometric failure (CIAF) and its determinants: A cross‐sectional study in the rural area of the Bogor District in Indonesia. BMC Nutrition, 8(1), 133. 36384860 10.1186/s40795-022-00627-3 PMC9666932

[fsn33821-bib-0034] Sabu, K. U. , Ravindran, T. S. , & Srinivas, P. N. (2020). Factors associated with inequality in composite index of anthropometric failure between the Paniya and Kurichiya tribal communities in Wayanad District of Kerala. Indian Journal of Public Health, 64(3), 258–265. 32985427 10.4103/ijph.IJPH_340_19 PMC7116138

[fsn33821-bib-0035] Seboka, B. T. , Hailegebreal, S. , Yehualashet, D. E. , & Demeke, A. D. (2021). Tracking progress in anthropometric failure among under‐five children in Ethiopia: A geospatial and multilevel analysis. Archives of Public Health, 79(1), 1–17. 34130742 10.1186/s13690-021-00615-2 PMC8207797

[fsn33821-bib-0036] Svedberg, P. (2000). Poverty and undernutrition: Theory, measurement, and policy. Clarendon Press.

[fsn33821-bib-0037] Udoh, E. E. , & Amodu, O. K. (2016). Complementary feeding practices among mothers and nutritional status of infants in Akpabuyo Area, Cross River State Nigeria. Springerplus, 5, 1–19. 28018781 10.1186/s40064-016-3751-7 PMC5138178

[fsn33821-bib-0038] UNICEF , WHO , & WB . (2020). Levels and trends in child malnutrition: Key findings of the 2019 Edition of the Joint Child Malnutrition Estimates .

[fsn33821-bib-0039] Walker, S. P. , Wachs, T. D. , Gardner, J. M. , Lozoff, B. , Wasserman, G. A. , Pollitt, E. , & Carter, J. A. (2007). Child development: Risk factors for adverse outcomes in developing countries. The Lancet, 369(9556), 145–157. 10.1016/S0140-6736(07)60076-2 17223478

[fsn33821-bib-0040] Wells, J. C. (2000). Natural selection and sex differences in morbidity and mortality in early life. Journal of Theoretical Biology, 202(1), 65–76. 10623500 10.1006/jtbi.1999.1044

[fsn33821-bib-0041] WHO . (2009). Infant and young child feeding: Model chapter for textbooks for medical students and allied health professionals. WHO Press. 23905206

[fsn33821-bib-0042] WHO . (2014). Global nutrition targets 2025: Stunting policy brief .

[fsn33821-bib-0043] WHO . (2017). Guideline: Assessing and managing children at primary health‐care facilities to prevent overweight and obesity in the context of the double burden of malnutrition . 29578661

[fsn33821-bib-0044] WHO . (2021). Indicators for assessing infant and young child feeding practices: Definitions and measurement methods .

[fsn33821-bib-0045] World Health Organization . (2019). Recommendations for data collection, analysis and reporting on anthropometric indicators in children under 5 years old .

[fsn33821-bib-0046] Ziba, M. , Kalimbira, A. A. , & Kalumikiza, Z. (2018). Estimated burden of aggregate anthropometric failure among Malawian children. South African Journal of Clinical Nutrition, 31(2), 20–23.

